# Whole body vibration and rider comfort determination of an electric two-wheeler test rig

**DOI:** 10.12688/f1000research.131105.3

**Published:** 2024-01-08

**Authors:** Keerthan Krishna, Sriharsha Hegde, Mahesha G T, Satish Shenoy B

**Affiliations:** 1 Department of Aeronautical and Automobile Engineering, Manipal Academy of Higher Education, Manipal, Karnataka, 576104, India

**Keywords:** Electric Two-wheeler, Rider comfort, Whole-body vibration, RMS Acceleration, Road profile

## Abstract

**Background:**

Two-wheeled vehicles are the major mode of transportation in India. Such vehicles are exposed to excessive vibration on the road when compared to four-wheeled vehicles. However, the research on the reduction of whole body vibration in the case of two-wheelers is not explored in detail. The present study predicts rider comfort in the case of an electric two-wheeler as per ISO 2631-1, by obtaining the finding the weighted acceleration at the strategic locations of vibration at the test rig.

**Methods:**

An electric two-wheeler test rig is used in the study. The values of acceleration from the test rig in running conditions are obtained by using NI LabVIEW 2019. The drive cycle of the electric vehicle (EV) test rig is controlled by Sync sols’ EV lab software. Obtaining the weighted root mean square (RMS) acceleration from running the test setup, it is compared with the ISO 2631-1 standard to obtain the rider comfort.

**Results:**

Loading area, traction motor, base mount, and suspension were found to be the strategic points of vibration. Frequency weighted RMS acceleration of 0.3 to 0.4 m/s ^2^ obtained at these points are prone to cause discomfort for the rider. Vehicle speed, road profile, and duration of exposure were found to be important parameters affecting the rider’s comfort. A maximum of 4.6 m/s ^2^ amplitude was observed. The loading area, which corresponds to a rider’s seat in actual vehicle, is important and reduction of these vibrations make the ride comfortable for the rider. Suspension and base mount of the test rig are found to be uncomfortable observing the weighted RMS acceleration.

**Conclusions:**

A suitable damping technique design is very much essential in reducing these vibrations and improve the rider comfort, as many more non-deterministic vibrations are prone to cause dis-comfort in case of actual on road riding conditions.

## Introduction

In India, the major mode of transportation is two-wheeled vehicles. ^1^ About 15 million two-wheeled vehicles were sold in India over the last 10 years on yearly basis. ^2^ Professionals like the food and goods delivery partners, low-wage employees, and post-delivery persons mainly use two-wheelers for their daily transportation. ^3^ Two-wheeled vehicles, owing to limited size and mass are prone to vibration when compared to four-wheeled vehicles. ^4^ Whole-body vibration (WBV) mainly affects these people who are in continuous exposure to noise and vibration throughout the day. ^5^^,^^6^ Truck drivers, drill operators, heavy machinery workers, and forklift drivers are the victims of these vibrations. ^7^ High risks of lower back pain, motion sickness, and digestive system problems have been reported due to WBV when exposed for a longer period. ^8^^,^^9^

The whole body vibration of the vehicle is dangerous not only to the rider but also to the vehicle as well. ^10^ In a vehicle exposed to different terrain conditions, the driving scenario is subjected to vibration, and these are transmitted to the human body through the seat, handlebar, and footrest in the case of two-wheelers. ^11^ These vibrations transferred to the human body cause different health issues in long run. ^12^^,^^13^ Some researchers are working on reducing these vibrations to effectively increase rider comfort. ^14^^–^^17^

A vehicle's comfort is influenced by many factors such as the seat design, ^18^ driving posture ^19^ and environmental factors, ^20^ road condition, and suspension system to name a few. In a two-wheeler, the rider’s comfort plays a very important role, as the rider has continuous exposure to these influencing factors. Both the static and dynamic condition of the vehicle are important in predicting the rider’s comfort. ^21^ Different kinds of shock absorbers, ^22^ and damping techniques ^23^ play a vital role in improving the rider’s comfort. Two-wheelers especially in the Indian scenario are very much subjected to vibration due to the condition of roads even in the cities. ^24^ The comfort level is greatly influenced by potholes, humps, cracks, and riding speed, and some study work has assisted in recognizing the potholes for safe driving. ^25^

The measurement of whole-body vibration in terms of human health and comfort, perception probabilities, and motion sickness occurrence is studied with the help of ISO 2631-1 standard. ^26^ It provides guidance on measurement techniques for periodic, random, and transient whole-body vibrations. ^27^ By getting the Frequency Response Function (FRF) at critical vibrational points, variable acceleration values at multiple spots were identified. The rider's comfort depends on these values of acceleration. The higher the value of acceleration lowers the rider's comfort. ISO 2631-1 standard provides different levels of comfort faced by riders depending on the acceleration values. Table 1 gives the detailed classification of the rider’s comfort level as per ISO 2631-1.

**Table 1.** Comfort level criteria. ^26^

Acceleration (m/s ^2^)	Category
Less than 0.315	Not uncomfortable
0.315-0.63	A little uncomfortable
0.5-1	Fairly uncomfortable
0.8-1.6	Uncomfortable
1.25-2.5	Very uncomfortable
Greater than 2.5	Extremely uncomfortable

The present study involves finding the strategic locations of vibration and evaluation of rider comfort on an electric two-wheeler (E2W) test rig. Different points at the test rig are evaluated for their acceleration values in different running conditions. The points (locations on the body of test rig) at which the amplitude of vibration is higher than other locations are considered strategic points of vibration. The vibration at the strategic points is high enough to cause discomfort to the rider. The impact hammer test ^28^ is conducted using the PCB (Pico Coulomb) Piezotronics made impact hammer of sensitivity 10.1 mV/g and data acquisition by using NI LabVIEW.

## Methods

### Development of state space model

The electric two-wheeler test rig is modeled as a state space model for finding the state space equations as shown in equation 1 and 2. Performing the impact hammer test on the setup, the natural frequencies are obtained. The details of the work carried out are discussed in this section.

Test setup

**Figure 1.**
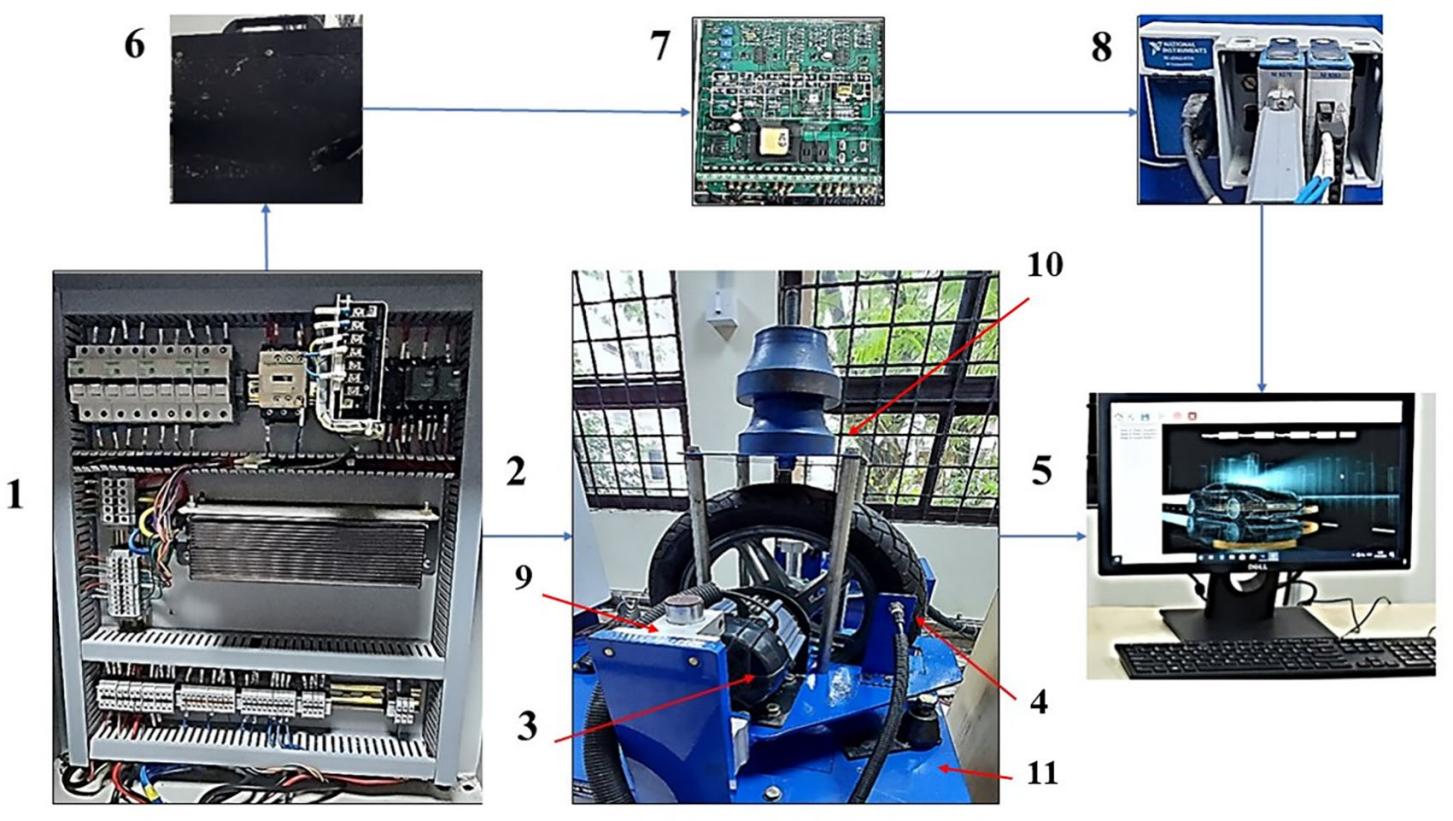
Electric two-wheeler test rig.

The test setup is an electric vehicle two-wheeler test rig, which uses a 1.5 kW, brushless direct current (BLDC) traction motor powered by a 25AH LiFePO _4_ battery. Figure 1 shows the photograph of different parts of the Electric two-wheeler (E2W) test rig (components sourced from Artis Technologies) and Table 2 gives the nomenclature.

**Table 2.** E2W test rig nomenclature.

Number	Part name
1	Electric panel
2	Wheel and the loading area
3	Traction motor
4	RPM sensor
5	Desktop with Sync sols EV lab Software
6	25 Ah Battery
7	Battery Modulator
8	NI Data Acquisition system
9	Suspension
10	Loading area
11	Base mount

**Figure 2.**
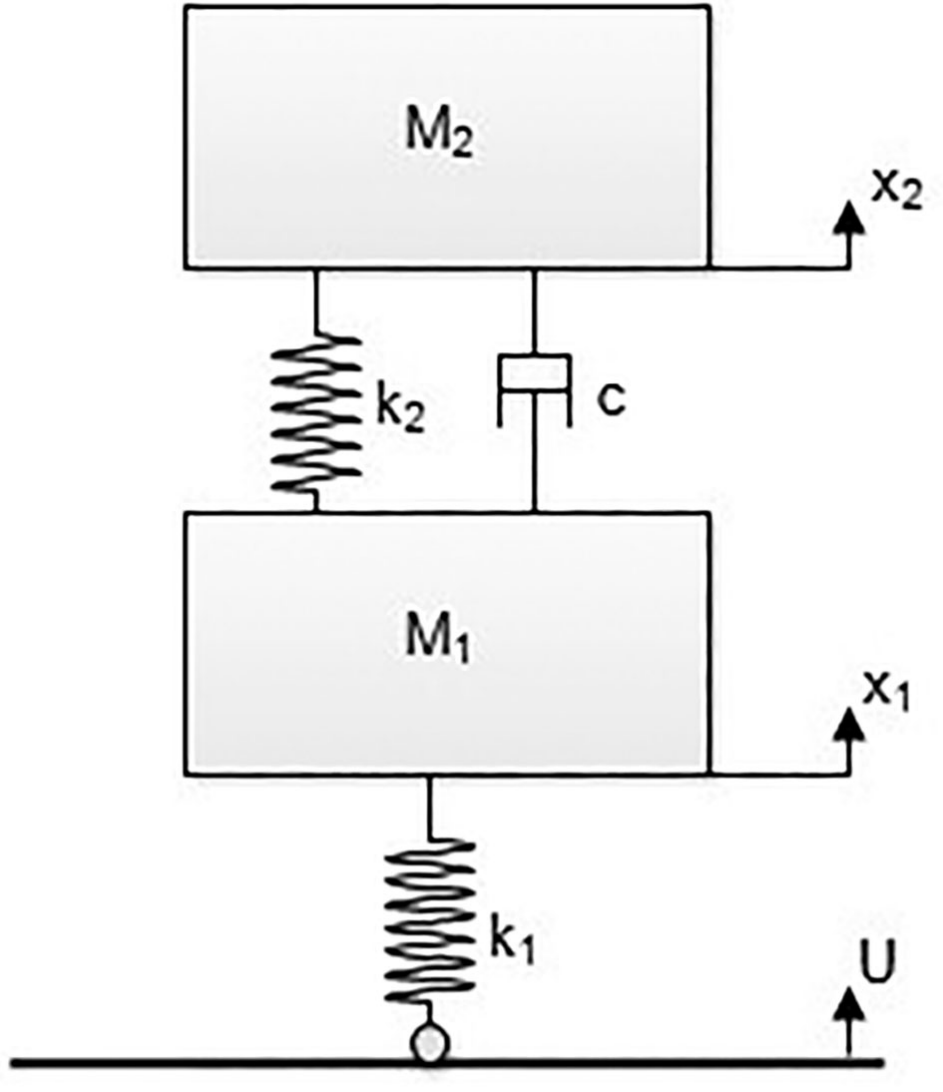
Model of the E2W test rig.

Using the laboratory's setup, the E2W test rig is modeled as state space model. ^29^ However, to build a similar model, two considerations were made, which are briefly discussed here. The first thing to consider is the cylindrical steel roller of approximately 40 mm diameter and 200 mm in length beneath the test rig's wheel is used to simulate a real-life road surface. The roller used is a hard plastic material in this case, however, unlike the road and wheel, the roller causes a small vertical displacement to the wheel. As a consequence, the vertical displacement of the roller concerning the vertical displacement of sprung and un-sprung masses is estimated to be near zero or zero. ^28^^,^^30^ The second subject of consideration concerns the sprung and un-sprung masses. Sprung mass is the percentage of the vehicle's overall mass that is supported by the suspension. Un-sprung mass refers to the mass of the suspension, wheels, and other components that are directly connected to them. This implies that a vehicle's sprung mass is typically the vehicle's kerb weight, the weight of the driver, and in certain cases, the weight of the engine. ^31^ A state space model of the E2W test rig is shown in Figure 2. ^32^ The model nomenclature is indicated in Table 3. The acceleration values are measured by the PCB Piezotronics made accelerometers of 101.1 mV/g sensitivity and data acquisition is carried out through National Instrument’s LabVIEW software. ( MyOpenLab is an open source alternative that can carry out a similar function). Three trials were conducted and the average values are considered for analysis.

**Table 3.** Test rig model nomenclature.

Variables	Description
M1	Un-sprung mass (tyre mass) – (kg)
M2	Sprung mass (Mass of test rig - Tyre mass) – (kg)
X1	Vertical displacement of M1 – (m)
X2	Vertical displacement of M2 – (m)
K1	Tyre stiffness – (N/m)
K2	Spring stiffness of vehicle suspension – (N/m)
C	Damping coefficient of the vehicle suspension – (Ns/m)
U	Vertical displacement of the roller – (m)

The free body and Laplace equations of the state space model derived are as indicated below in equations 1, 2 and 3, 4 respectively:

$$m_{2}{\ddot{x}}_{2}+c\;\left({\dot{x}}_{2}–{\dot{x}}_{1}\right)+k_{2}\;\left(x_{2}–x_{1}\right)=0$$ (1)

$$m_{1}{\ddot{x}}_{1}+c\;\left({\dot{x}}_{1}–{\dot{x}}_{2}\right)+k_{2}\;\left(x_{1}–x_{2}\right)+k_{1}\left(x_{1}-U\right)=0$$ (2)

Laplace equations

$$X_{2}\left(s\right)\;\left[M_{2}S^{2}+\mathrm{CS}+K_{2}\right]=X_{1}\left(s\right)\;\left[\mathrm{CS}+K_{1}\right]$$ (3)

$$X_{1}\left(s\right)\;\left[M_{1}S^{2}+\mathrm{CS}+K_{2}+K_{1}\right]=X_{2}\left(s\right)\;\left[\mathrm{CS}+K_{2}\right]+U\left(s\right)\;\left[K_{1}\right]$$ (4)

Figure 2 shows the state space model of the E2W test rig and the corresponding notations as indicated in the figure. Here, the governing system equation of the test rig ( 1 & 2) are derived to get the Laplace equations ( 3 & 4).

**Figure 3.**
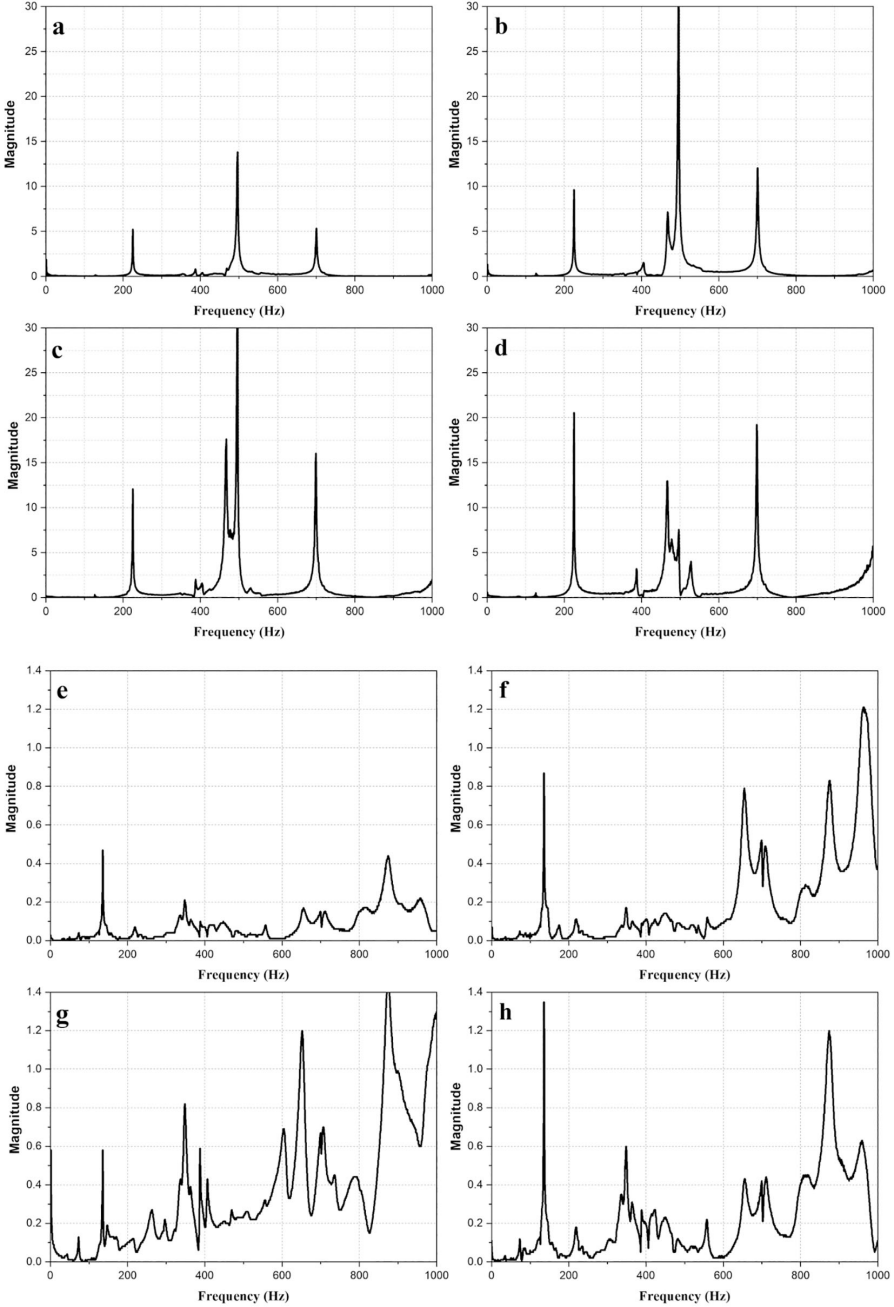
Impact hammer test on E2W test rig (a,b,c,d - Loading area- left back, right back, left front, right front), (e,f,g,h – Base mount – left back, right back, left front, right front).

Figure 3 shows the magnitude vs frequency plots of the impact hammer test conducted on the E2W test rig. The plot obtained from the NI LabVIEW, 2019 ^33^ shows the natural frequency of the test rig as obtained at two strategic points on the test rig as shown in Figure 3. The peaks in the graphs indicated the natural frequency of the rig. The average of the natural frequency obtained is shown in Table 4.

**Table 4.** Average of natural frequencies.

Frequency (Hz)	Magnitude (g)
180.225	6.338
575.36	11.37
786.942	7.03

### Obtaining acceleration using LabVIEW

National Instruments’ LabVIEW 2019 (64-bit) software is used to extract the acceleration values through accelerometers along with conversion of raw acceleration values into RMS acceleration. Some open software like ‘MyOpenLab’ or PyLab_Works can be used for data acquisition as well. Fast Fourier transform (FFT) ^34^ is used to obtain the root mean square (RMS) acceleration values at the strategic locations. Using LabVIEW, the RMS acceleration is obtained using spectral analysis provided in the software. The values are then converted to frequency weighted RMS acceleration as per ISO 2631-1 standard. The acceleration in z-axis is considered and ‘wk’ weighting factor is considered.

The test setup is tested under different loading conditions such as kerb load, 5 kg load, and 10 kg load. This type of loading makes a machine or a material get stiffer as the load increases. ^35^ PCB Piezotronics made accelerometers are mounted at four strategic locations of vibration as indicated in Figure 1; the loading area, traction motor, suspension and the base mount of the rig. RMS acceleration, at these strategic locations, is recorded using LabVIEW programming. These values are then sorted using MS Excel to find the peak values at each interval and graphs are plotted to show the RMS acceleration vs frequency characteristics as shown in Figure 5.

No load condition of the electric two-wheeler test rig is conducted without adding any payload. This condition reveals the strategic location of the test rig and allows identifying the major vibration amplitude regions. Adding payload of 5 kg and 10 kg the mechanical vibration characteristics of the test rig changes showing different vibration patterns. This pattern of vibrations is studied in order to obtain the dangerous frequencies and amplitudes. Strategic locations especially the loading area, corresponds to rider’s seat in an actual two-wheeler and hence ISO 2631-1 standard is compared in this study.

Drive cycle

**Figure 4.**
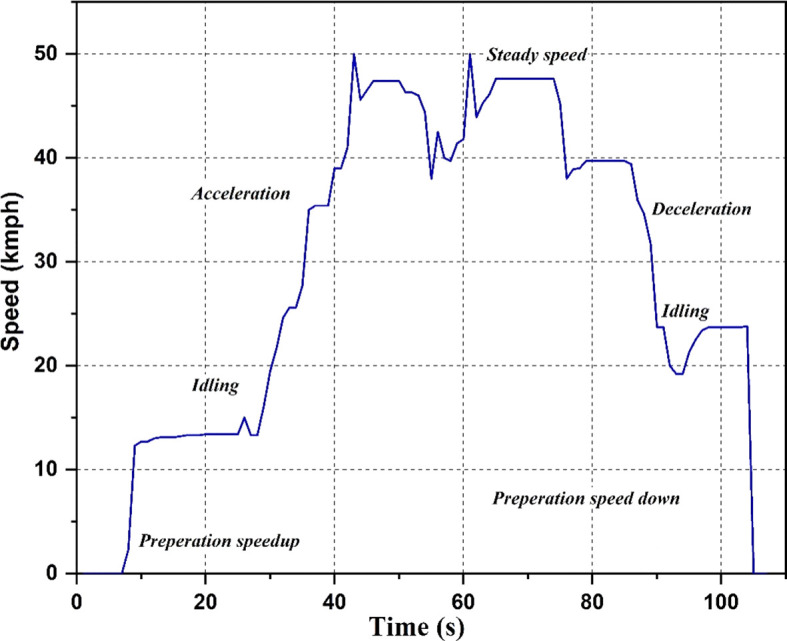
Drive Cycle.

The drive cycle used for the study is shown in Figure 4. Different scenarios like idling, acceleration, steady speed, and deceleration are shown in the graph. The drive cycle runs each of these scenarios for a particular time duration. The cycle begins with a preparation speed-up period of 5 seconds, followed by 20 seconds of idling, 18 seconds of acceleration, and 2 seconds of steady speed. The cycle then decelerates for the next 11 seconds, a combination of acceleration and steady speed for the next 7 seconds, decelerates for the next 30 seconds, idles for the next 11 seconds, and ends with 3 seconds of halting. The values of acceleration are recorded at the strategic locations during this cycle.

## Results and discussion

In this study, major strategic locations of vibrations in an electric two-wheeler test rig are found. The vibration response from the strategic locations indicates the rider’s comfort through weighted RMS acceleration values. As compared to ISO 2631-1, the results obtained are discussed in detail in this section.

**Figure 5.**
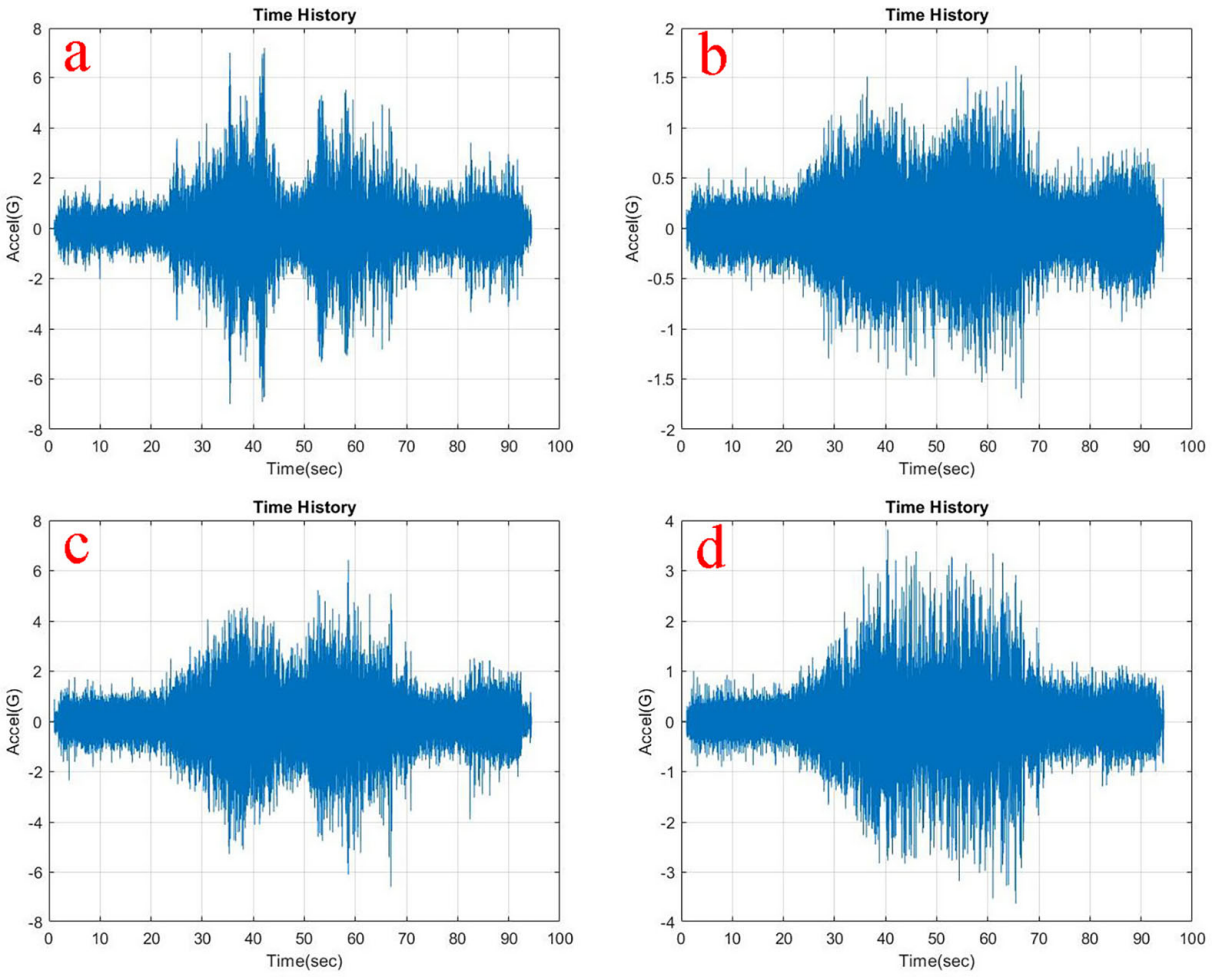
RMS Acceleration at the strategic locations (a) loading area, b) traction motor, c) suspension, d) base mount) at no load condition.

**Figure 6.**
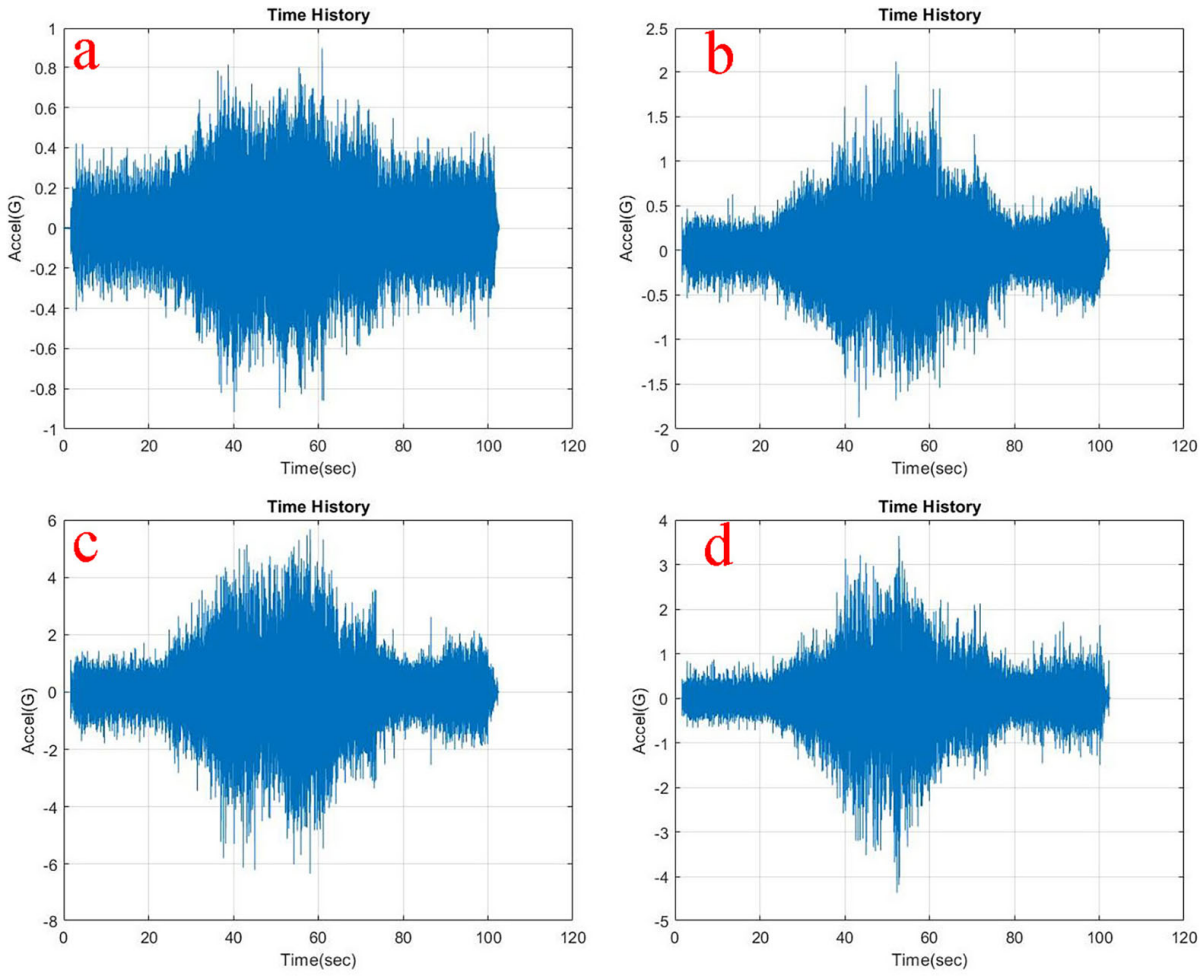
RMS accelerations at the strategic locations (a) loading area, b) traction motor, c) suspension, d) base mount) at 5 kg loading.

**Figure 7.**
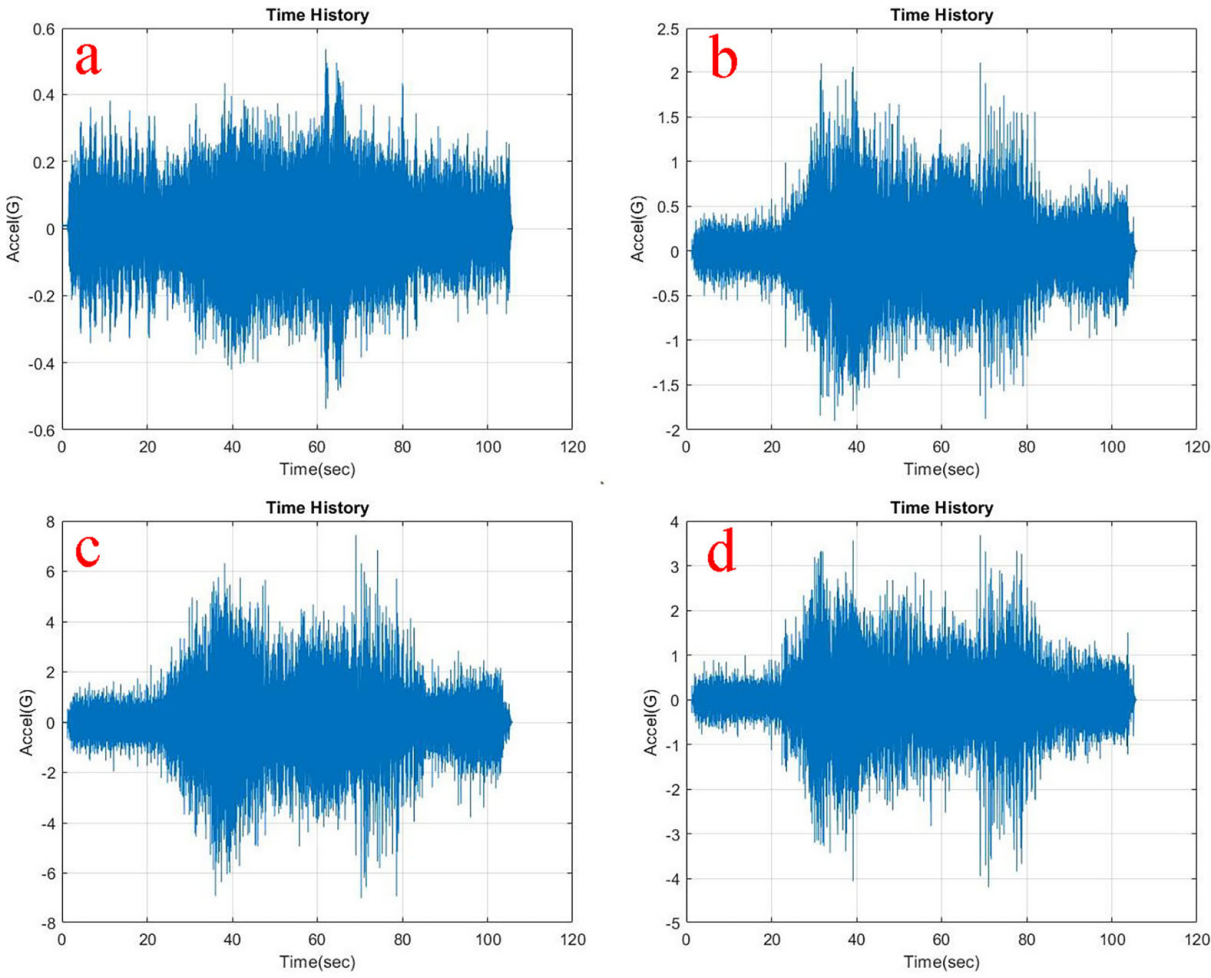
RMS accelerations at the strategic locations (a) loading area, b) traction motor, c) suspension, d) base mount) at 10 kg loading.

Observing, the weighted acceleration at the loading area as well as suspension as shown in Figures 5, 6 and 7 it is noted that, the acceleration response at the loading area is near to the uncomfortable acceleration as guided by ISO 2631-1 standard as shown in Table 1. Upon loading of 5 kg the vibration amplitudes increased by about 5.8% and 2.2% at loading area and suspension respectively. This increase is observed due to the change in loading condition and stiffness as shown in the state space model. However, upon loading 10 kg the response measured indicates slightly lesser vibration characteristics showing about 4.3% and 3% at loading area and suspension respectively.

At the traction motor as well as base mount of the test rig it can be observed from Figures 5, 6 and 7 that, the vibration decreased upon increasing the load from no load to 5 kg and further to 10 kg. About 7.27% and 8.91% of weighted acceleration was decreased by the addition of 5 kg and about 11.1% and 18.93% decrement in weighted acceleration is observed when 10 kg loading is added to the system at traction motor and base mount of the rig respectively. This clearly signifies the mass damping phenomenon in the system when the loads are added.

**Figure 8.**
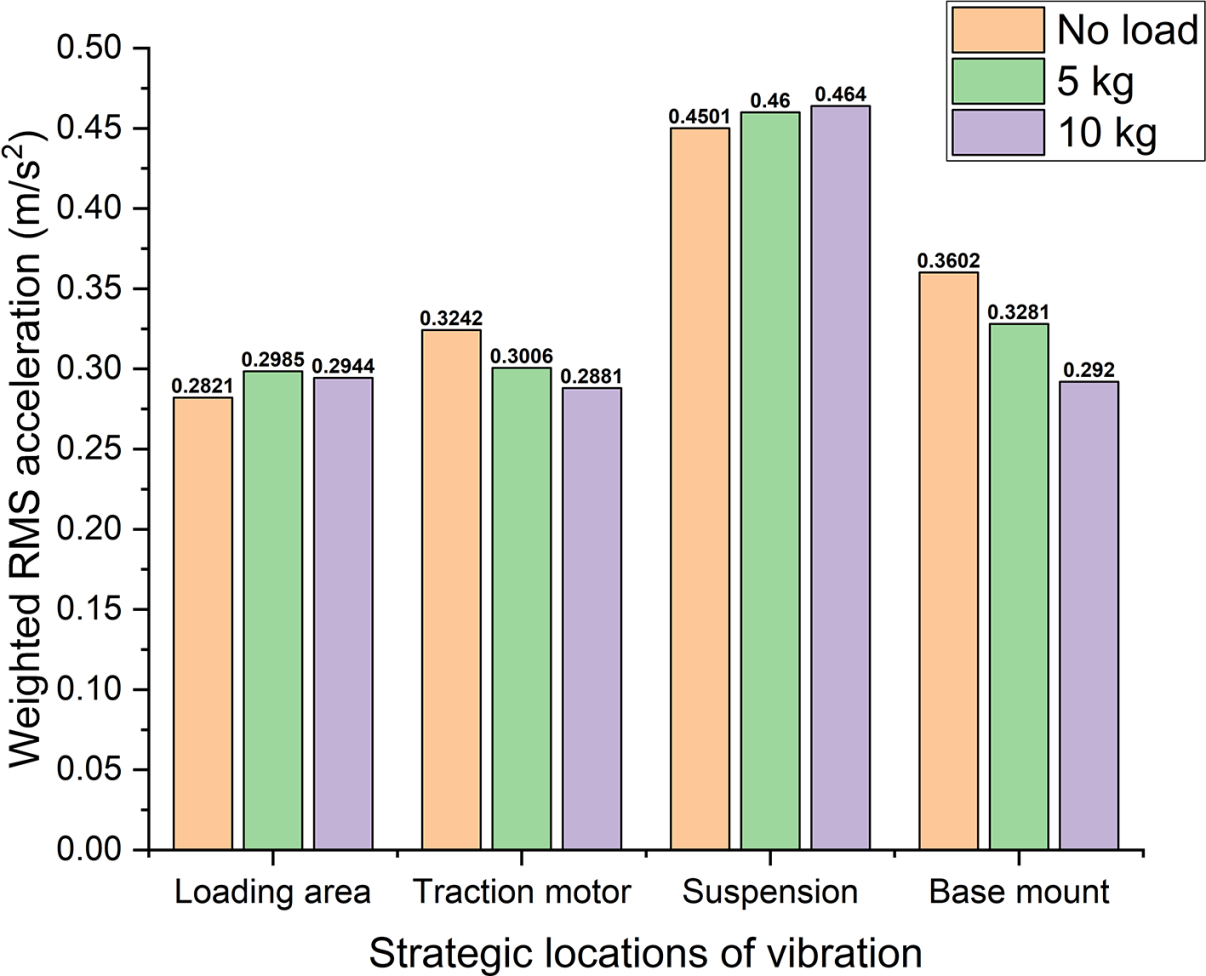
Comparison of weighted acceleration at strategic locations of the test rig.

With reference to Table 1, Figure 8 shows the weighted acceleration comparison at different strategic locations of vibrations in the electric two-wheeler test rig. Observing the rms acceleration at the loading area it shows ~0.29 m/s ^2^ of maximum RMS acceleration. This shows that this location is very close to the uncomfortable region with reference to ISO 2631-1 standard. However, at the traction motor, suspension, and base mount, the rms acceleration values indicate them to be under uncomfortable region. Here uncomfortable region indicates that, these vibrations are prone to cause dis-comfort for the rider when an actual two-wheeler is considered. Hence, these vibrations need to be addressed with proper damping systems to enhance the rider’s comfort.

**Figure 9.**
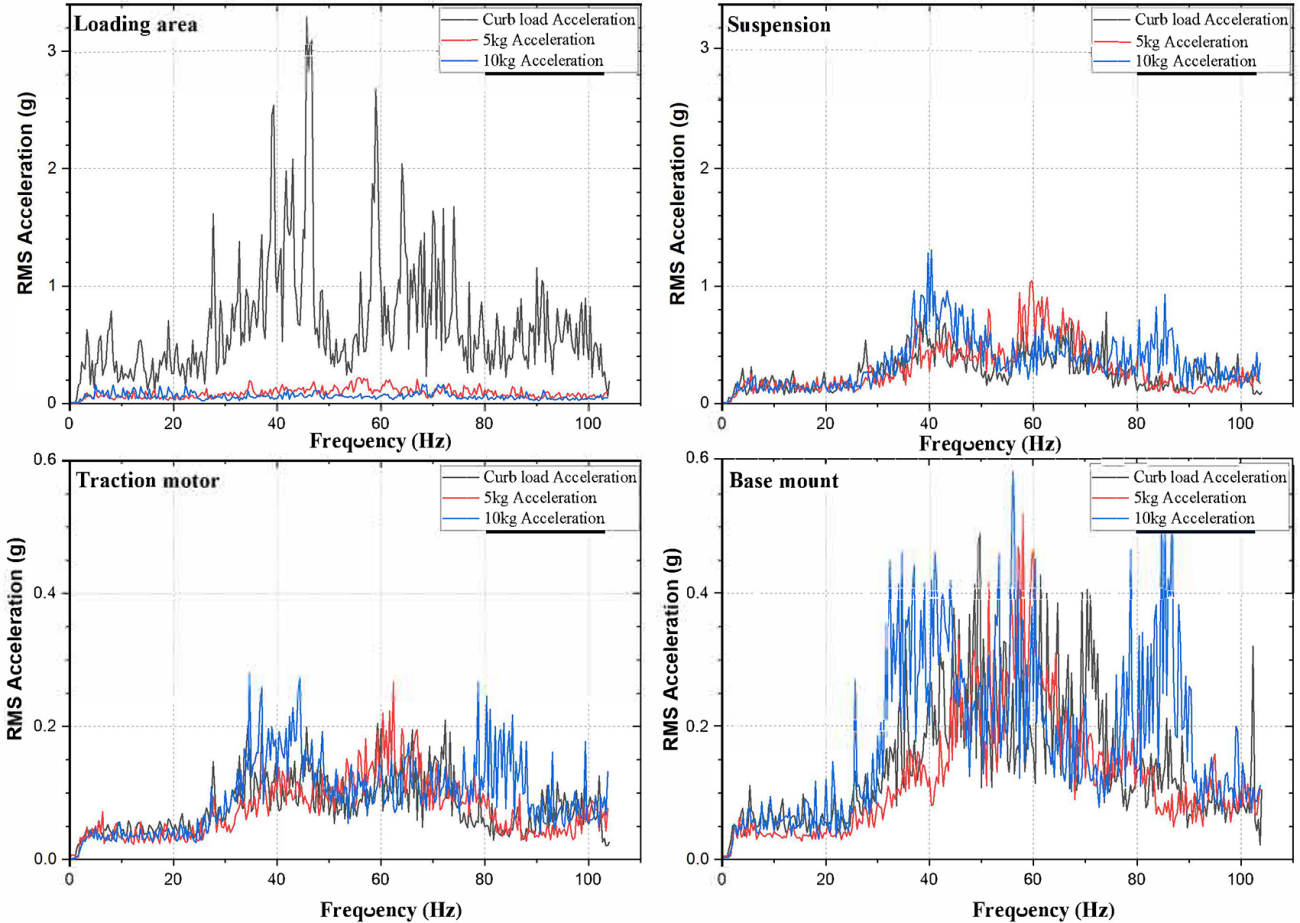
Exciting frequencies at the strategic locations.

Another important criterion to be considered to evaluate the rider’s comfort are the excitation frequencies. ISO 2631-1 standard focusses on the frequencies from 0.5 Hz to 80 Hz in evaluating the WBV and human comfort. In this regard, Figure 9 shows the excitation frequencies in the E2W test rig. These frequencies observed are well under the ISO criterion and it is observed to affect the rider’s comfort. From the Figure 9 it can be seen that, Loading area which corresponds to the seat of the actual vehicle has several exciting frequencies within the range of 0.5 Hz to 80 Hz and these are prone to cause dis-comfort for the rider.

## Conclusions

A detailed experimental analysis of finding the strategic locations of vibration is discussed in this paper. The acceleration values play an important role in deciding the rider’s comfort. Electric two-wheeler, even though a cost-effective mode of transportation, require further research in improving rider comfort. Observing the different strategic locations of vibration at the test rig the following conclusions are drawn from this study.

Observing different strategic locations i.e., loading area, traction motor, suspension, and base mount of the test rig, the vibrations generated at these key points affect the rider’s comfort. Comparing the weighted acceleration values with the ISO 2631-1 standard, the vibrations generated are found to be at uncomfortable region at the key locations of the test rig. The addition of weights to the system showed a decrement in vibration levels which is due to the phenomenon of mass damping. Further, exciting frequencies at the test rig shows several frequency components that affect the rider’s comfort. Hence, a suitable damping treatment is always essential to reduce these affects on the human body.

However, as the speed increases, the vibration intensity increased as well. This is due to the wheels running on the roller support, which simulates an actual road scenario. Hence, it can be concluded that in the actual driving scenario of a two-wheeler the vibration increases as the speed of the vehicle is increased. Further, the condition of the road is again an influencing factor, which increases the vibration intensity.

Overall study indicates that the electric two-wheeler is subjected to vibrations is an important area to be considered for further research work and this can be reduced by using suitable damping techniques at the strategic locations of vibrations.

## Data Availability

Figshare: data for paper submitted to f1000 research. https://doi.org/10.6084/m9.figshare.22092101.v1. ^36^ This project contains the following underlying data:
‐ 5kg raw data fig 4.xlsx (RMS acceleration for 5kg loading) ‐ 10kg raw data fig 4.xlsx (RMS acceleration for 10kg loading) ‐ Down left back fig 3e.xlsx (Impact hammer test data at base mount at back side left) ‐ Down left front fig 3g.xlsx (Impact hammer test data at base mount at front side left). ‐ Down right back fig 3f.xlsx (Impact hammer test data at base mount at back side right). ‐ Down right front fig 3g.xlsx (Impact hammer test data at base mount at front side right). ‐ No load raw data fig 4.xlsx (RMS acceleration for No load condition). ‐ Raw Data for fig 5.lvm (RMS acceleration obtained at strategic locations). ‐ Top left back fig 3c.xlsx (Impact hammer test data at loading area at back side left). ‐ Top right back fig 3b.xlsx (Impact hammer test data at loading area at back side right). ‐ Top right front 3d.xlsx (Impact hammer test data at loading area at front side right). Data are available under the terms of the Creative Commons Zero “No rights reserved” data waiver (CC0 1.0 Public domain dedication).
