# Supplementary material for: Whole body vibration and rider comfort determination of an electric two-wheeler test rig
Source: F1000Res. 2024 Oct 30;12:559. Originally published 2023 May 30. [Version 5] doi: 10.12688/f1000research.131105.5 (PMC11134141; doi:10.12688/f1000research.131105.5)
Supplement: Peer review history

# Reviewer report: Reviewer response for version 5

**DOI:** 10.5256/f1000research.173971.r336542

**Published:** 2024-11-07

**Authors:** Roberto Ventura, Benedetto Barabino

**Affiliations:** 1 Università degli Studi di Brescia, Brescia, Italy; 2 DICATAM, Universita degli Studi di Brescia (Ringgold ID: 9297), Brescia, Lombardy, Italy

The authors did not address our previous report and ignored all of our comments.

Is the work clearly and accurately presented and does it cite the current literature?

Partly

If applicable, is the statistical analysis and its interpretation appropriate?

Not applicable

Are all the source data underlying the results available to ensure full reproducibility?

Yes

Is the study design appropriate and is the work technically sound?

Partly

Are the conclusions drawn adequately supported by the results?

Partly

Are sufficient details of methods and analysis provided to allow replication by others?

Partly

Reviewer Expertise:

Micromobility, Transportation Safety, Risk Modelling.

We confirm that we have read this submission and believe that we have an appropriate level of expertise to confirm that it is of an acceptable scientific standard, however we have significant reservations, as outlined above.

# Reviewer report: Reviewer response for version 3

**DOI:** 10.5256/f1000research.160573.r273316

**Published:** 2024-08-01

**Authors:** Roberto Ventura, Benedetto Barabino

**Affiliations:** 1 Università degli Studi di Brescia, Brescia, Italy; 2 DICATAM, Universita degli Studi di Brescia (Ringgold ID: 9297), Brescia, Lombardy, Italy

This study investigates the whole-body vibration of two-wheeled vehicles by performing some experimental trials on a two-wheeler test rig. Acceleration data are processed according to ISO 2631-1 standard to compute weighted RMS values. Hence, RMS values are compared with ISO 2631-1 thresholds to infer the expected comfort level faced by riders.

 While the paper explores an interesting topic deserving attention, its contribution to the current state of the art lacks clarity. Moreover, some technical aspects must be refined.

**Comment 1**

 Background

 Although the riding comfort of two-wheeled vehicles is a well-researched topic, this aspect is surprisingly only marginally evident in the literature review provided. The authors seem to have overlooked several crucial previous studies, both theoretical and experimental in nature.

 On the theoretical front, for instance, Cano Moreno et al. [Ref-1,5] conducted a comprehensive analytical assessment of the impact of e-scooter vibrations on driver comfort and health through simulations. Additionally [Ref-2] evaluated motorcycle comfort levels using a frequency-domain approach, considering human sensitivity, and highlighted the dependence of proposed comfort indices on suspension characteristics.

 Turning to the experimental realm, [Ref-3,4] performed empirical studies comparing the comfort experienced by riders of e-bikes and e-kick scooters in terms of vibrational acceleration. These studies conducted real-world trials across various road surfaces and user demographics. Notably, they found that e-kick scooters generally induce higher vibrational magnitudes than e-bikes. Furthermore, factors such as pavement surface, sensor position, user gender, height, and travel speed emerged as critical determinants of vibrational magnitude for both vehicles.

 It is strongly recommended that the authors enrich their literature review by incorporating these seminal studies along with other relevant contributions. Such inclusion would provide a more comprehensive understanding of the state-of-the-art research in this field and offer valuable insights for advancing their own work.

**Comment 2**

 Contributions

 It's crucial to highlight the unique contributions and distinctions of the current study from existing literature. This involves identifying gaps in the current research landscape that the study addresses. The authors should clearly outline how their work stands out from the current state of the art, whether through innovative methodologies or fresh perspectives. Justifying the necessity of the study is essential, demonstrating its significance in advancing knowledge or addressing critical issues. Additionally, any methodological innovations introduced should be succinctly described to underscore their relevance and potential impact.

**Comment 3**

 Materials and methods

 The authors should clarify the criteria used to select a load range between 5 kg and 10 kg. This load range may seem relatively light, particularly when compared to the average user weight of 60-80 kg. Additionally, the study only considers acceleration in the z-axis and the ISO 2631-1 "wk" weighting factor. This limitation is significant since real-world scenarios involve transversal accelerations caused by factors such as braking and turning manoeuvres. Consequently, concerns arise about the applicability of the test rig experiment results to real-world conditions. It would be beneficial for the authors to justify this choice, ensuring it aligns with realistic usage conditions and enhances the credibility and relevance of the study's findings. Finally, it would be useful if the authors could provide an estimation of the parameters governing the analytical model of the test rig (i.e., M1, M2, K1, K2, C).

**Comment 4**

 Discussion of the results

 In a scientific paper, it is essential to discuss the results by comparing them with the findings of previous work to identify similarities and inconsistencies. For instance, is the observed positive correlation between speed and vibration intensity an expected outcome? Similarly, is the influence of road conditions an expected finding? To the best of my knowledge, it is. Please provide further discussion.

**Comment 5**

 Conclusions

 The conclusions provided in the manuscript partially lack elaboration on the research's theoretical and practical implications, limitations, and future research perspectives. It is essential to delve deeper into these aspects to provide a comprehensive understanding of the study's significance and potential contributions to the field.

Is the work clearly and accurately presented and does it cite the current literature?

Partly

If applicable, is the statistical analysis and its interpretation appropriate?

Not applicable

Are all the source data underlying the results available to ensure full reproducibility?

Yes

Is the study design appropriate and is the work technically sound?

Partly

Are the conclusions drawn adequately supported by the results?

Partly

Are sufficient details of methods and analysis provided to allow replication by others?

Partly

Reviewer Expertise:

My primary research expertise includes the safety of infrastructures subjected to heavy vehicle transit, risk analyses in the transportation field, and the dynamics of micromobility vehicles.

We confirm that we have read this submission and believe that we have an appropriate level of expertise to confirm that it is of an acceptable scientific standard, however we have significant reservations, as outlined above.

## References

[1] : E-scooter Vibration Impact on Driver Comfort and Health. *Journal of Vibration Engineering & Technologies* .2021;9(6) : 10.1007/s42417-021-00280-3 1023-1037 10.1007/s42417-021-00280-3

[2] : Frequency-domain method for evaluating the ride comfort of a motorcycle. *Vehicle System Dynamics* .2006;44(4) : 10.1080/00423110500420712 339-355 10.1080/00423110500420712

[3] : First experimental comparison between e-kick scooters and e-bike’s vibrational dynamics. *Transportation Research Procedia* .2022;62: 10.1016/j.trpro.2022.02.092 743-751 10.1016/j.trpro.2022.02.092

[4] : Comparing the vibrational behaviour of e-kick scooters and e-bikes: Evidence from Italy. *International Journal of Transportation Science and Technology* .2023; 10.1016/j.ijtst.2023.10.010 10.1016/j.ijtst.2023.10.010

[5] : Methodology for the study of the influence of e-scooter vibrations on human health and comfort. In Proceedings of the Seventh International Conference on Technological Ecosystems for Enhancing Multiculturality. *Research gate* . 10.1145/3362789.3362906 10.1145/3362789.3362906

# Reviewer report: Reviewer response for version 3

**DOI:** 10.5256/f1000research.160573.r231721

**Published:** 2024-05-28

**Authors:** Bui Van Cuong

**Affiliations:** 1 Automotive and Power Machinery Engineering, Thai Nguyen University of Technology, Thai Nguyen, Vietnam

I appreciate the research contribution of the author team. However, the study has several limitations and only partially addresses each aspect. Here are some suggestions for improvement:

 1) The inconsistency in capitalization in Table 3 and Figure 2 should be rectified.

 2) Authors should use symbols like "m _s_" and "m _u_" for Sprung mass and Unsprung mass, respectively.

 3) Specific parameters are missing in Table 3.

 4) The method for determining the Root-Mean-Square (RMS) value of acceleration in Figure 8 needs clarification. Which equation?

 5) The frequency range used is not appropriate for ISO 2631-1 evaluation. It is crucial to analyze the low-frequency range from 1-15Hz, particularly the frequency band sensitive to human perception, which lies between 4-8Hz. This adjustment would ensure a more accurate assessment of vibration effects on human comfort.

 6) Equations 1 to 4 on page 6 are presented but not simulated. To enhance content quality, numerical simulations should be conducted alongside experiments.

Is the work clearly and accurately presented and does it cite the current literature?

Partly

If applicable, is the statistical analysis and its interpretation appropriate?

Partly

Are all the source data underlying the results available to ensure full reproducibility?

Partly

Is the study design appropriate and is the work technically sound?

Partly

Are the conclusions drawn adequately supported by the results?

Partly

Are sufficient details of methods and analysis provided to allow replication by others?

Partly

Reviewer Expertise:

My research interests encompass vehicle dynamics and control, optimal design and control methodologies, as well as active and semi-active control strategies for suspension system. I also specialize in multi-objective optimization techniques, including Genetic Algorithms (GA) and Particle Swarm Optimization (PSO).

I confirm that I have read this submission and believe that I have an appropriate level of expertise to confirm that it is of an acceptable scientific standard.

# Response

**Published:** 2024-06-07

**Authors:** Satish Shenoy B

**Affiliations:** Manipal University, India

We are very grateful for the reviewer’s comments and all the necessary changes have been incorporated in the manuscript and highlighted.

**Reviewer: Bui Van Cuong, Automotive and Power Machinery Engineering, Thai Nguyen University of Technology, Thai Nguyen, Vietnam.**

**Comments:**

**1. The inconsistency in capitalization in Table 3 and Figure 2 should be rectified.**

**Answer: The inconsistency in capitalization has been rectified and updated in the paper.**

**2. Authors should use symbols like "ms" and "mu" for Sprung mass and Unsprung mass, respectively.**

**Answer: Currently m1 and m2 is used for sprung mass and Unsprung mass. However, this will be taken care of further in the research papers.**

**3. Specific parameters are missing in Table 3.**

**Answer: Table 3 has been updated accordingly.**

**4. The method for determining the Root-Mean-Square (RMS) value of acceleration in Figure 8 needs clarification. Which equation?**

**Answer: The method of obtaining RMS acceleration is explained in “Obtaining acceleration using LabVIEW” section of the paper. Here, the RMS value is found through LabVIEW software as mentioned, and not through equations.**

**5. The frequency range used is not appropriate for ISO 2631-1 evaluation. It is crucial to analyze the low-frequency range from 1-15Hz, particularly the frequency band sensitive to human perception, which lies between 4-8Hz. This adjustment would ensure a more accurate assessment of vibration effects on human comfort.**

**Answer: Low-frequency zone is highlighted in the figure and updated in Figure 9. However, as ISO 2631-1 states the assessment of vibration in the range of 0.5 Hz – 80 Hz, this figure is adopted in the paper. Further, in the research, this frequency region will be considered.**

**6. Equations 1 to 4 on page 6 are presented but not simulated. To enhance content quality, numerical simulations should be conducted alongside experiments.**

**Answer: This would be taken as a future scope of the paper and will be done as per suggestions.**

**Thank you**

# Reviewer report: Reviewer response for version 3

**DOI:** 10.5256/f1000research.160573.r236174

**Published:** 2024-01-30

**Authors:** Peter Múčka

**Affiliations:** 1 Slovak Academy of Sciences, Bratislava, Slovakia

Manuscript was partially improved. I consider the scientific level or contribution of the results to be still limited. The article is more of a measurement report than a scientific article. The influence of the mass change on the vibration of the quarter-car model is well known.

Is the work clearly and accurately presented and does it cite the current literature?

No

If applicable, is the statistical analysis and its interpretation appropriate?

Partly

Are all the source data underlying the results available to ensure full reproducibility?

Partly

Is the study design appropriate and is the work technically sound?

No

Are the conclusions drawn adequately supported by the results?

No

Are sufficient details of methods and analysis provided to allow replication by others?

No

Reviewer Expertise:

Vibration, vehicle dynamics, signal processing

I confirm that I have read this submission and believe that I have an appropriate level of expertise to confirm that it is of an acceptable scientific standard.

# Reviewer report: Reviewer response for version 2

**DOI:** 10.5256/f1000research.155303.r209188

**Published:** 2023-10-03

**Authors:** Peter Múčka

**Affiliations:** 1 Slovak Academy of Sciences, Bratislava, Slovakia

Manuscript was partially improved but several comments were ignored.

- The results do not offer any substantial new results in area of two-wheeled vehicle vibration.
- Reviewer did not consider this paper to be the contribution to the topic of two-wheeled vehicles comfort.
- All presented results or dependences are known (sprung mass increase influence on vibration).
- The comparison with ISO 2631-1 reaction levels is inappropriate because the ISO 2631-1 uses frequency-weighted acceleration.
- Typical natural frequencies of suspended quarter-car model are about 1 Hz for sprung mass and 10 Hz for unsprung mass.
- The important frequency range in ISO 2631-1 is up to 80 Hz.
- The article lacks the discussion of natural frequencies of the test rig and its influence on tested vehicle vibration with the published literature.

Specific comments:

 Page 4

 “The electric two-wheeler test rig is modeled as a state space model for finding its natural frequencies.”

 COMMENT: Natural frequencies of measuring system were identified experimentally by impact hammer. The connection between quarter-car model (Fig. 2) parameters and identified natural frequencies was not presented.

 Page 7 – Fig. 3

 COMMENT: Add the dimension to the Magnitude.

 Page 8

 “Hence, comparing these values with ISO 2631 as given in table 1, the comfort category has moved to comfortable region from extremely uncomfortable region after loading.” …

 “As compared to ISO 2631, the results obtained are discussed in detail in this section.”

 COMMENT: As it was stated in first review, it is not appropriate to compare the frequency-weighted specified in ISO 2631-1 and unweighted acceleration values.

 Page 9 – Fig. 6

 COMMENT: Using 6 decimal points for frequency is superfluous.

Is the work clearly and accurately presented and does it cite the current literature?

No

If applicable, is the statistical analysis and its interpretation appropriate?

Partly

Are all the source data underlying the results available to ensure full reproducibility?

Partly

Is the study design appropriate and is the work technically sound?

No

Are the conclusions drawn adequately supported by the results?

No

Are sufficient details of methods and analysis provided to allow replication by others?

No

Reviewer Expertise:

Vibration, vehicle dynamics, signal processing

I confirm that I have read this submission and believe that I have an appropriate level of expertise to state that I do not consider it to be of an acceptable scientific standard, for reasons outlined above.

# Response

**Published:** 2023-12-23

**Authors:** Satish Shenoy B

**Affiliations:** Manipal University, India

**Rebuttal to Reviewer comments**

 We are very grateful for the reviewer’s comments and all the necessary changes have been incorporated in the manuscript.

**General comments:**

**1. Manuscript was partially improved but several comments were ignored.**

**Answer:** Manuscript is updated with all the comments addressed.

**2. The results do not offer any substantial new results in area of two-wheeled vehicle vibration.**

**Answer:** The results have been improved by adding frequency weighted acceleration. Finding this acceleration and comparing the results with ISO 2631-1 standard the rider’s comfort is accessed and this is a value addition to the field.

**3. Reviewer did not consider this paper to be the contribution to the topic of two-wheeled    vehicles comfort.**

**Answer: **Kindly find the new manuscript update in which a detailed explanation is provided.

**4. All presented results or dependences are known (sprung mass increase influence on vibration).**

**Answer: **The study is mainly focused on finding the strategic locations of vibrations which affect the rider’s comfort. These points are found to mitigate these issues in future. The sprung and unsprung mass are indicated as to find the state space equations.

**5. The comparison with ISO 2631-1 reaction levels is inappropriate because the ISO 2631-1 uses frequency-weighted acceleration**

**Answer: **Frequency weighted acceleration is considered and updated with appropriate results and discussion.

**6. Typical natural frequencies of suspended quarter-car model are about 1 Hz for sprung mass and 10 Hz for unsprung mass.**

**Answer: **Here, the impact hammer test reveals the natural frequency of the entire electric two-wheeler test rig.

**7. The important frequency range in ISO 2631-1 is up to 80 Hz.** **Answer: **Yes, ISO 2631-1 suggests that frequency range of 0.5-80 Hz. However, these frequencies affecting the human body are an ongoing work and this paper doesn’t include the same. This paper deals with understanding the rider’s comfort level in accordance with the weighted acceleration comparison with ISO standards. However, the natural frequency study is beyond the scope of this paper.

**8. The article lacks the discussion of natural frequencies of the test rig and its influence on tested vehicle vibration with the published literature.**

**Answer: **The article has been updated with discussion on the peak frequencies affecting the rider’s comfort. This study is mainly focused on finding rider’s comfort and hence only the frequencies under the range of 0.5 Hz to 80 Hz is considered as per ISO 2631-1.

**Specific comments:**

**1. Fig 4 “The electric two-wheeler test rig is modeled as a state space model for finding its natural frequencies.”** **COMMENT: Natural frequencies of measuring system were identified experimentally by impact hammer. The connection between quarter-car model (Fig. 2) parameters and identified natural frequencies was not presented.**

**Answer: **The sentence has been modified and updated.

**2. Page 7 – Fig. 3** **COMMENT: Add the dimension to the Magnitude.**

**Answer: **The magnitude in the y-axis represents the impact of the hammer on the machine and it is dimensionless.

**3. Page 8 “Hence, comparing these values with ISO 2631 as given in table 1, the comfort category has moved to comfortable region from extremely uncomfortable region after loading.” …“As compared to ISO 2631, the results obtained are discussed in detail in this section.”** **COMMENT: As it was stated in first review, it is not appropriate to compare the frequency-weighted specified in ISO 2631-1 and unweighted acceleration values.**

**Answer: **The updated paper includes weighted acceleration comparison with ISO 2631-1, and I hope now it adds value to the comparison.

**4. Page 9 – Fig. 6** **COMMENT: Using 6 decimal points for frequency is superfluous.**

**Answer: **It has been modified and updated.

# Reviewer report: Reviewer response for version 1

**DOI:** 10.5256/f1000research.143915.r187825

**Published:** 2023-08-16

**Authors:** Le Van Quynh

**Affiliations:** 1 Automotive and Power Machinery Engineering, Thai Nguyen University of Technology, Thai Nguyen City, Vietnam

Thank you for your work and the paper submitted. There is a lot of work presented and I would suggest some revisions to improve the quality of the paper

- Most of the analysis results, the authors only reconstructed considering the value analysis on the time domain based on ISO 2631-1 standard, but did not analyze the frequency domain, especially the low frequency domain. Therefore, the goal of smooth analysis is not vehicle ride comfort. I advise the authors to correct and add to clarify.
- Page 4: The author shows the measuring positions in Fig.1.
-  Page 5: Eq.(1)- E(4) have not been found to be related to the experimental results, please review?
-  Page 8: the y axis representation of Fig.5 is the acceleration values, not RMS acceleration values, please review?

Is the work clearly and accurately presented and does it cite the current literature?

Partly

If applicable, is the statistical analysis and its interpretation appropriate?

Partly

Are all the source data underlying the results available to ensure full reproducibility?

Partly

Is the study design appropriate and is the work technically sound?

Partly

Are the conclusions drawn adequately supported by the results?

Partly

Are sufficient details of methods and analysis provided to allow replication by others?

Partly

Reviewer Expertise:

Vibration Analysis; Nonlinear Dynamic Structural; Vibration Modal

I confirm that I have read this submission and believe that I have an appropriate level of expertise to confirm that it is of an acceptable scientific standard, however I have significant reservations, as outlined above.

# Response

**Published:** 2023-08-29

**Authors:** Satish Shenoy B

**Affiliations:** Manipal University, India

We are very grateful for the reviewer’s comments and all the necessary changes have been incorporated in the manuscript.

 Please note all the comments are addressed in the paper and highlighted for reference.

**2. Le Van Quynh, Automotive and Power Machinery Engineering, Thai Nguyen University of Technology, Thai Nguyen City, Vietnam**

**General comments:**

- **Most of the analysis results, the authors only reconstructed considering the value analysis on the time domain based on ISO 2631-1 standard, but did not analyze the frequency domain, especially the low frequency domain. Therefore, the goal of smooth analysis is not vehicle ride comfort. I advise the authors to correct and add to clarify.**

**Answer:** The graphs and plots showing frequency domain have been added to the paper (Figure 5) and explained.

- **Page 4: The author shows the measuring positions in Fig.1.**

**Answer: **All the positions along with strategic locations have been updated in the figure.

  

- **Page 5: Eq.(1)- E(4) have not been found to be related to the experimental results, please review?**

**Answer: **Here the equations 1 & 2 are the governing system equations and later derived Laplace equations (3 & 4) represent the state space equations. The detail explanation is again added to the manuscript.

  

- **Page 8: the y axis representation of Fig.5 is the acceleration values, not RMS acceleration values, please review?**

**Answer:** RMS acceleration have been indicated and it is updated in version 2 of the paper.

# Reviewer report: Reviewer response for version 1

**DOI:** 10.5256/f1000research.143915.r187826

**Published:** 2023-07-24

**Authors:** Peter Múčka

**Affiliations:** 1 Slovak Academy of Sciences, Bratislava, Slovakia

The work is not clearly and accurately presented. It cites the current literature.

 The academic merit of the work is low.

 The novelty of the work is limited. This is the report from vibration measurement.

 Details of methods and analysis provided do not allow replication by others. The interpretation of results is confused (the measured values are not comparable in principle with ISO 2631-1 comfort reaction levels).

 In my opinion the work does not offer the novel or important results.

 General comments:

 1. ISO 2631-1 does not specify impact hammer test approach. Please clarify.

 2. Page 3 – Methods

 COMMENT: State-space representation is a mathematical model of a physical system related by first-order (not involving second derivatives) differential equations. The used quarter-car model (Fig. 2) for two-wheeler test rig is described by differential equations involving second derivative. Clarify.

 3. Authors measured the acceleration at two-wheeled test rig. It is not clear why the quarter-car model (Fig. 2), equations of motions (1-4) and Table 3 Test rig model nomenclature are used in the paper. I did not see any results connected with them.

 4. Page 7 “Obtaining acceleration using LabVIEW“

 COMMENT: This approach should be connected and explained with definitions in ISO 2631-1.

 Where is the measuring point?  ISO 2631-1 specified the likely reactions of passengers in public transport. Where is passenger in your case?

 5. Page 8 “These values are then compared with the ISO 2631-1 standard to further analyze the rider comfort.”

 COMMENT: ISO 2631-1 is specified for standing, sitting and recumbent persons. ISO 2631 used the frequency-weighted acceleration.  Clarify.

 6. Page 8 – Comparison of measured acceleration values at the test rig with comfort level criteria based on frequency-weighted acceleration measured at sitting, standing or recumbent persons is not correct.

 7. The results are not compared with observations and results of other authors. What is the impact of such results?

 Specific comments:

 Page 3 – “By getting the Frequency Response Function (FRF) at critical vibrational points, variable acceleration values at multiple spots were identified”

 COMMENT: ISO 2631-1 did not specify this approach for acceleration identification. Clarify.

 Page 3. “Table 1 gives the detailed classification of the rider’s comfort level as per ISO 2631-1.”

 COMMENT: Specify how acceleration was estimated in line with ISO 2631-1: 1997.

 Annex C.2.3 Comfort reactions to vibration environments in ISO 2631-1 specified magnitudes of overall vibration total values.

 Page 5 – Eq. (2)

 COMMENT: In Eq. (2) the variable x is used for kinematic excitation (vertical excitation of the rolller) but the Fig. 2 used the variable U.

 Page 5, Table 2

 COMMENT: C (Ns/m) is not damping ratio but damping coefficient.

 Is this ok? “M1 Un-sprung mass (tyre mass) – (kg)”.

 I assume, the first dof (X1) ist he roller mass (undamped) vibration and the second dof (X2) is tyre mass (damped) vibration. Please clarify.

 Page 6, Figure 3

 COMMENT: The Scheme of Impact hammer test on E2W test rig (cases a) to h) may be visualized. It is not clear what is “loading area” and what is “base mount”.

 Page 7

 "The values of acceleration are recorded at the strategic locations during this cycle.“

 COMMENT: Where are these strategic locations? Clarify.

 Page 7

 COMMENT: Add the scheme of location of PCB Piezotronics accelerometers.

 Page 7

 “Table 4. Average of natural frequencies.”

 COMMENT: Natural frequencies of what system? Match the natural frequencies to the particular parts of analysed mechanical system.

Is the work clearly and accurately presented and does it cite the current literature?

No

If applicable, is the statistical analysis and its interpretation appropriate?

Partly

Are all the source data underlying the results available to ensure full reproducibility?

Partly

Is the study design appropriate and is the work technically sound?

No

Are the conclusions drawn adequately supported by the results?

No

Are sufficient details of methods and analysis provided to allow replication by others?

No

Reviewer Expertise:

Vibration, vehicle dynamics, signal processing

I confirm that I have read this submission and believe that I have an appropriate level of expertise to state that I do not consider it to be of an acceptable scientific standard, for reasons outlined above.

# Response

**Published:** 2023-08-29

**Authors:** Satish Shenoy B

**Affiliations:** Manipal University, India

We are very grateful for the reviewer’s comments and all the necessary changes have been incorporated in the manuscript.

 Please note all the comments are addressed in the paper and highlighted for reference.

1. **Peter Múčka, Slovak Academy of Sciences, Bratislava, Slovakia**

***General comments:***

**1. ISO 2631-1 does not specify impact hammer test approach. Please clarify.**

**Answer:** Impact hammer test was conducted in order to check if any operating frequencies co-inside with the natural frequency of the system. Accordingly, one of the natural frequencies (180Hz) was found to near to the natural frequency of the system and the relation has been mentioned and updated in the conclusions.

**2. Page 3 – Methods**

**COMMENT: State-space representation is a mathematical model of a physical system related by first-order (not involving second derivatives) differential equations. The used quarter-car model (Fig. 2) for two-wheeler test rig is described by differential equations involving second derivative. Clarify.**

**Answer:** Here the equations 1 & 2 are the governing system equations and later derived Laplace equations (3 & 4) represent the state space equations. The detail explanation is again added and updated to the manuscript.

**3. Authors measured the acceleration at two-wheeled test rig. It is not clear why the quarter-car model (Fig. 2), equations of motions (1-4) and Table 3 Test rig model nomenclature are used in the paper. I did not see any results connected with them.**

**Answer: **The equations 1 to 4 are derived to show the system mathematically and this is basic study conducted on the test rig. The research has continued considering an actual vehicle and converting the ICE vehicle to Electric as well. Understanding the quarter car model of the test rig is important in understanding the dynamics of the system.

**4. Page 7 “Obtaining acceleration using LabVIEW“**

**COMMENT: This approach should be connected and explained with definitions in ISO 2631-1.**

**Where is the measuring point?  ISO 2631-1 specified the likely reactions of passengers in public transport. Where is passenger in your case?**

**Answer: **Strategic locations have been indicated and have been updated in the version 2 of the paper. However, ISO 2631 standard is compared in this paper against the RMS acceleration obtained at the strategic locations. As these strategic locations especially the loading area corresponds to the rider’s seat in an actual two-wheeler, this standard has been used. Further, this study is continued in an actual two-wheeler considering an actual rider and road interactions. Which is the future scope of the this paper.

**5. Page 8 “These values are then compared with the ISO 2631-1 standard to further analyze the rider comfort.”**

**COMMENT: ISO 2631-1 is specified for standing, sitting and recumbent persons. ISO 2631 used the frequency-weighted acceleration.  Clarify.**

**Answer: **Here ISO 2631 is compared with the RMS acceleration obtained at the strategic locations of vibration. As these strategic locations especially the loading area corresponds to the rider’s seat in an actual two-wheeler, this standard has been used.

**6. Page 8 – Comparison of measured acceleration values at the test rig with comfort level criteria based on frequency-weighted acceleration measured at sitting, standing or recumbent persons is not correct.**

**Answer: **Frequency weightings not considered in this paper. This paper only compares the RMS acceleration to determine the peak amplitudes in the test rig. Frequency weightings and calculations of Vibration dose value is the future scope of this paper.

**7. The results are not compared with observations and results of other authors. What is the impact of such results?**

**Answer: **We have not come across such results from literatures. These results taken from accelerometers in an electric two-wheeler test rig and these results are unique.

**Specific comments:**

**Page 3 – “By getting the Frequency Response Function (FRF) at critical vibrational points, variable acceleration values at multiple spots were identified”** **COMMENT: ISO 2631-1 did not specify this approach for acceleration identification. Clarify.**

**Answer: **Conducting FRF is a process conducted using NI’s LabVIEW software. This is conducted in order to convert the raw acceleration obtained to RMS acceleration at the strategic locations. Obtaining RMS acceleration, later these values are compared with ISO 2631 as mentioned in the standard. ISO 2631 specifies comfort categories based on RMS acceleration, and only this part of the standard is being compared with the results obtained.

**Page 3. “Table 1 gives the detailed classification of the rider’s comfort level as per ISO 2631-1.”** **COMMENT: Specify how acceleration was estimated in line with ISO 2631-1: 1997.**

**Annex C.2.3 Comfort reactions to vibration environments in ISO 2631-1 specified magnitudes of overall vibration total values.**

**Answer: **These have been added in the version 2 adding RMS accelerations which signifies the overall vibration total values as specified by the standard.

**Page 5 – Eq. (2)** **COMMENT: In Eq. (2) the variable x is used for kinematic excitation (vertical excitation of the rolller) but the Fig. 2 used the variable U.**

**Answer: **These variables are identified and rectified at both the locations. The roller excitation is indicated as U.

**Page 5, Table 2** **COMMENT: C (Ns/m) is not damping ratio but damping coefficient.**

**Is this ok? “M1 Un-sprung mass (tyre mass) – (kg)”.**

**I assume, the first dof (X1) is the roller mass (undamped) vibration and the second dof (X2) is tyre mass (damped) vibration. Please clarify.**

**Answer: **The variable name is identified and rectified. C is damping coefficient. Here X1 is the vertical displacement of mass M1 and X2 is the vertical displacement of Mass M2.

**Page 6, Figure 3** **COMMENT: The Scheme of Impact hammer test on E2W test rig (cases a) to h) may be visualized. It is not clear what is “loading area” and what is “base mount”.**

**Answer: T**he strategic locations i.e. loading area, traction motor, suspension and base mount are updated in figure 1 in version 2 of the paper.

**Page 7** **"The values of acceleration are recorded at the strategic locations during this cycle.“**

**COMMENT: Where are these strategic locations? Clarify.**

**Answer: **The strategic locations are as shown in figure 1 (loading area, traction motor, suspension and base mount).

**Page 7** **COMMENT: Add the scheme of location of PCB Piezotronics accelerometers.**

**Answer: **Scheme of location of PCB Piezotronics accelerometers are added to the paper and highlighted for reference.

**Page 7** **“Table 4. Average of natural frequencies.”**

**COMMENT: Natural frequencies of what system? Match the natural frequencies to the particular parts of analysed mechanical system.**

**Answer: **Here the natural frequencies shown are of the entire electric two-wheeler test rig. The frequency is matched with particular part in version 2 of the paper.
